# Negative binomial mixture model for identification of noise in antibody-antigen specificity predictions from single-cell data

**DOI:** 10.1093/bioadv/vbae170

**Published:** 2024-12-04

**Authors:** Perry T Wasdin, Alexandra A Abu-Shmais, Michael W Irvin, Matthew J Vukovich, Ivelin S Georgiev

**Affiliations:** Program in Chemical and Physical Biology, Vanderbilt University Medical Center, Nashville, TN, 37232, United States; Center for Computational Microbiology and Immunology, Vanderbilt University Medical Center, Nashville, TN, 37232, United States; Vanderbilt Vaccine Center, Vanderbilt University Medical Center, Nashville, TN, 37232, United States; Vanderbilt Vaccine Center, Vanderbilt University Medical Center, Nashville, TN, 37232, United States; Department of Pathology, Microbiology and Immunology, Vanderbilt University Medical Center, Nashville, TN, 37232, United States; Multiscale Modeling Group, Computational Biology Hub, Altos Laboratories, Redwood City, CA, 94065, United States; Vanderbilt Vaccine Center, Vanderbilt University Medical Center, Nashville, TN, 37232, United States; Department of Pathology, Microbiology and Immunology, Vanderbilt University Medical Center, Nashville, TN, 37232, United States; Program in Chemical and Physical Biology, Vanderbilt University Medical Center, Nashville, TN, 37232, United States; Center for Computational Microbiology and Immunology, Vanderbilt University Medical Center, Nashville, TN, 37232, United States; Vanderbilt Vaccine Center, Vanderbilt University Medical Center, Nashville, TN, 37232, United States; Department of Pathology, Microbiology and Immunology, Vanderbilt University Medical Center, Nashville, TN, 37232, United States; Department of Computer Science, Vanderbilt University, Nashville, TN, 37232, United States; Vanderbilt Institute for Infection, Immunology and Inflammation, Vanderbilt University Medical Center, Nashville, TN, 37232, United States; Center for Structural Biology, Vanderbilt University, Nashville, TN, 37232, United States

## Abstract

**Motivation:**

LIBRA-seq (linking B cell receptor to antigen specificity by sequencing) provides a powerful tool for interrogating the antigen-specific B cell compartment and identifying antibodies against antigen targets of interest. Identification of noise in single-cell B cell receptor sequencing data, such as LIBRA-seq, is critical for improving antigen binding predictions for downstream applications including antibody discovery and machine learning technologies.

**Results:**

In this study, we present a method for denoising LIBRA-seq data by clustering antigen counts into signal and noise components with a negative binomial mixture model. This approach leverages single-cell sequencing reads from a large, multi-donor dataset described in a recent LIBRA-seq study to develop a data-driven means for identification of technical noise. We apply this method to nine donors representing separate LIBRA-seq experiments and show that our approach provides improved predictions for *in vitro* antibody-antigen binding when compared to the standard scoring method, despite variance in data size and noise structure across samples. This development will improve the ability of LIBRA-seq to identify antigen-specific B cells and contribute to providing more reliable datasets for machine learning based approaches as the corpus of single-cell B cell sequencing data continues to grow.

**Availability and implementation:**

All data and code are available at https://github.com/IGlab-VUMC/mixture_model_denoising.

## 1 Introduction

**Figure 1.**
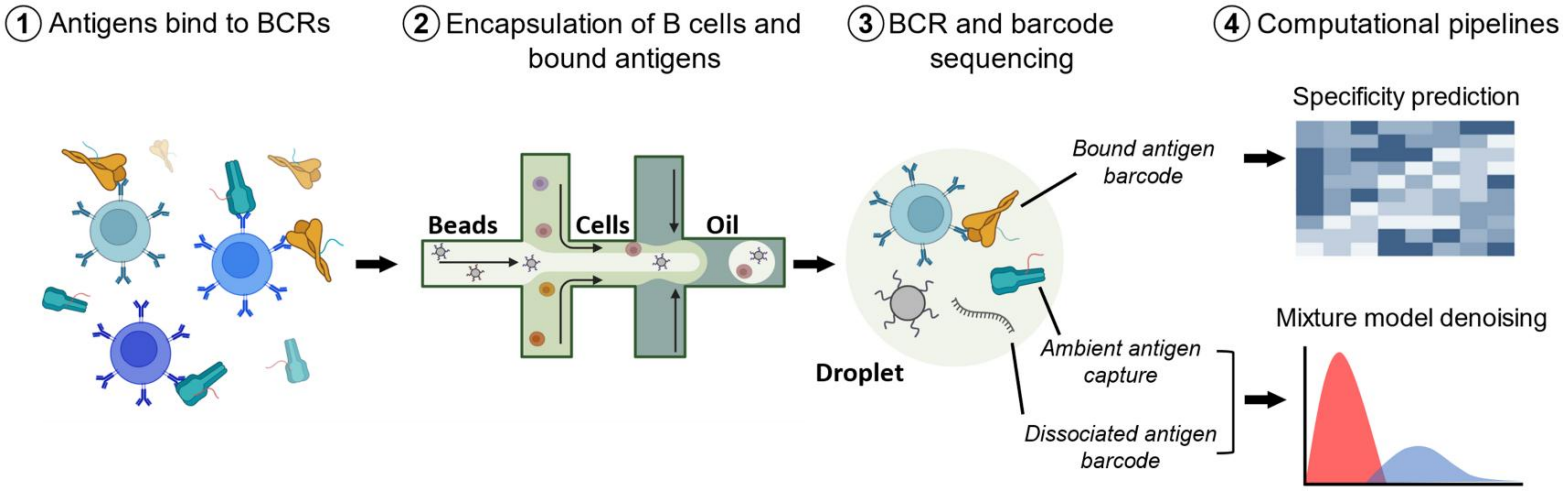
Basic schematic of single cell capture in LIBRA-seq and potential sources of noise in UMI values for an antigen: (1) Barcoded antigens are mixed with donor PBMCs, then antigen-positive B cells are sorted out for sequencing. (2) Droplet encapsulation of single cells and bound antigens. (3) Ambient barcode and antigen capture within droplet lead to false positive UMI counts. (4) Computational pipelines are used to analyze output from antigen barcode sequencing including the established LIBRA-seq pipeline for predicting BCR specificity and the pipeline presented here to remove noise based on ambient antigen and dissociated antigen barcode capture.

B cells are a critical component of the adaptive immune system. They bind to foreign antigens via the B cell Receptor (BCR) in order to form immunological memory or differentiate into antibody-secreting plasma cells (Inoue and Kurosaki 2024). The B cell compartment within humans is highly diverse, resulting in a repertoire of BCRs capable of exquisite specificity for an almost unlimited number of antigens. The identification of antigen-specific B cells is of interest for the development of vaccines and monoclonal antibody therapeutics, and also provides a basis for improving our understanding of the role of BCR diversity in the adaptive immune response to infection and vaccination. LIBRA-seq (linking B cell receptor to antigen specificity by sequencing) provides a powerful tool for interrogating the antigen-specific B cell compartment and identifying antibodies against antigen targets of interest (Setliff *et al.* 2019). In this technology (Fig. 1), a panel of antigens are labeled with oligonucleotide barcodes and then mixed with donor peripheral blood mononuclear cells (PBMCs). Antigen-bound B cells are then isolated and sequenced, enabling high-throughput mapping of single B cells to their cognate antigens.

In single-cell sequencing (using the 10x Genomics platform), each molecule in a droplet following the encapsulation of cells (step 2 in Fig. 1) is labelled with a unique oligonucleotide molecular identifier (UMI) tag (Zheng *et al.* 2017). In the following discussion, UMI counts refer to the UMIs associated with the barcodes used to tag each antigen type in the LIBRA-seq study. The standard LIBRA-seq bioinformatic pipeline uses these UMIs to calculate LIBRA-seq scores (LSS), which represent binding between an antigen and a BCR, based on the number of antigen barcodes associated with each droplet. LSS are calculated by applying a centered log-ratio transform followed by a Z-score transformation to provide relative scores which can be compared across cells for each antigen within a sample. This transformation has been shown to be effective for identification of antigen-specific B cells, but LSS can fail to account for noise due to inflated UMI counts in some LIBRA-seq datasets, resulting in false positive errors for binding predictions. While denoising methods have been published for other multi-modal single cell sequencing methods, these may not be subjected to the same technical biases as LIBRA-seq and the use of these denoising approaches has not been demonstrated in the LIBRA-seq context (Gayoso *et al.* 2021, Mulè *et al.* 2022). It will therefore be of value to develop a lightweight approach for deconvoluting noise from signal in the LIBRA-seq outputs, especially in order to false positive errors for improved downstream applications and antibody discovery.

The denoising approach described here aims to address two major sources of experimental noise which can lead to the superficial inflation of antigen UMI counts (Fig. 1, step 3). First, barcodes could dissociate between the final purification step during sample processing and sequencing, resulting in ambient barcodes which are encapsulated in droplets without their antigen. Second, antigens could bind to a B cell or BCR by non-specific binding due to hydrophobic patches, here referred to as “sticky” antigens. While these UMI counts may reflect interaction between a B cell and antigen, they likely do not reflect the highly specific binding interactions which are of interest for therapeutic applications. It is also possible that free-floating antigens in solution could be ambiently captured without interacting with the BCR at all. We do not attempt to differentiate between these processes here, rather we are attempting to identify UMI counts resulting from any sources of technical noise that may be present.

Despite these limitations, LIBRA-seq and similar methods have proven to be immensely useful for antibody discovery applications (Kramer *et al.* 2022, Shiakolas *et al.* 2022, Walker *et al.* 2022, Pilewski *et al.* 2023), and are also quickly becoming a valuable source of antibody-antigen specificity data for computational applications, which has, until recently, been infeasible to collect in a high-throughput manner. While large databases of antibody sequences exist (Abanades *et al.* 2024, Raybould *et al.* 2021, Olsen *et al.* 2022), there are currently no antigen-specific antibody databases of adequate size and scope for machine learning applications, and synthetic data often must be incorporated (Akbar *et al.* 2022). The available body of LIBRA-seq data continues to grow, promising to become an important source of data for future technologies aimed at predicting antigen specificity. However, the usefulness of this data for such applications hinges on the reliability of binding predictions. Aside from machine learning applications, further experimental validation of antigen binding predictions from LIBRA-seq is expensive and time consuming, and the field stands to benefit greatly from improved accuracy of antigen specificity predictions from single-cell BCR sequencing-based technologies.

## 2 Methods

### 2.1 Datasets

A recent study from our group aimed to generate large-scale antibody-antigen datasets, detailing the collection and characterization of LIBRA-seq data for a set of 10 healthy donors (samples) across a panel of 20 antigens (Abu-Shmais *et al.* 2024). This study provides a rich dataset for development of the denoising model presented here. In the LIBRA-seq experiments, an antigen screening library of 20 antigens from diverse but common pathogens were included. In addition, cells from the Ramos cell line which were engineered to express the HIV-1-specific antibody VRC01(Zhou *et al.* 2010) were also included, along with an HIV-1 antigen (BG505), to serve as negative controls: since the donors were screened for HIV-1, they theoretically should have very few antibodies which bind to the BG505 antigen, while the VRC01 expressing cells should only bind to the BG505 antigen and none of the other antigens in the LIBRA-seq screening library. In our approach, we leveraged these negative control HIV-1 specific cells by assuming that UMI counts associated with VRC01 cells and non-HIV antigens represent technical noise in the experiment. As a result, the VRC01 cells provide information about the structure of noise for each antigen in each sample and could be used to bias our denoising algorithm. *In vitro* validation data was also collected for 99 antibodies of interest, using their BCR sequences to recombinantly express the antibodies and test for binding against their identified cognate antigen using ELISA (enzyme-linked immunosorbent assays).

#### 2.1.1 Preprocessing

In the Atlas experiments, PBMCs from each donor were processed and sequenced as individual samples. Raw sequencing reads from 10× single cell RNA- and VDJ-sequencing were aligned to the human reference genome GRCh20 (2020) using Cell Ranger 3.1.0. Calculation of LSS was performed using the LIBRA-seq bioinformatic pipeline, as previously described. In the standard pipeline, UMI counts <4 are typically set to 0 before the LSS transformation, as UMI counts this low are considered unreliable. For the denoising approach presented here, these UMI counts were retained so that the count distributions would accurately reflect the structure of noise and signal captured in the experiments. Cells associated with more than one heavy chain (*N* > 1) were removed before denoising as these cells could potentially represent sequencing errors or multiplet capture. For each sample, VRC01 cells were separated from the real donor cells by comparing the heavy chain CDR3 sequences to the VRC01 sequence with a Levenshtein distance threshold of 0.05 (representing 95% identity). UMI counts >99th percentile were assumed to be outliers and were removed from the VRC01 and donor B cell distributions.

Cell counts following this preprocessing are summarized in Supplementary Table S1 for nine donors chosen to be used in the development and application of our denoising approach here. The total number of donor B cells recovered ranged from 160 B cells in Donor 5 to 4100 B cells in Donor 1. The number of negative control cells recovered ranged from 0 cells in Donor 3 to 6766 cells in Donor 6. Donor 2 was chosen to be used for example visualizations in the Section 3 as a substantial number of both donor and VRC01 cells were recovered, but other donors are included in the figures and supplement as applicable. Despite the broad antigen panel used in the experiments, SARS-CoV-2 spike was the only antigen consistently recovered in high numbers across donors. An average of 486 UMI counts were recovered for SARS-CoV-2 across all donors, compared to an average of less than 50 cells with >10 UMI counts for all other antigens. Additionally, some donors from the original set of experiments were excluded based on low cell counts (<50 total) and overall poor sample quality. Further insight into the necessary data size for this approach is included later in the discussion.

#### 2.1.2 Characterization of noise in data

**Figure 2.**
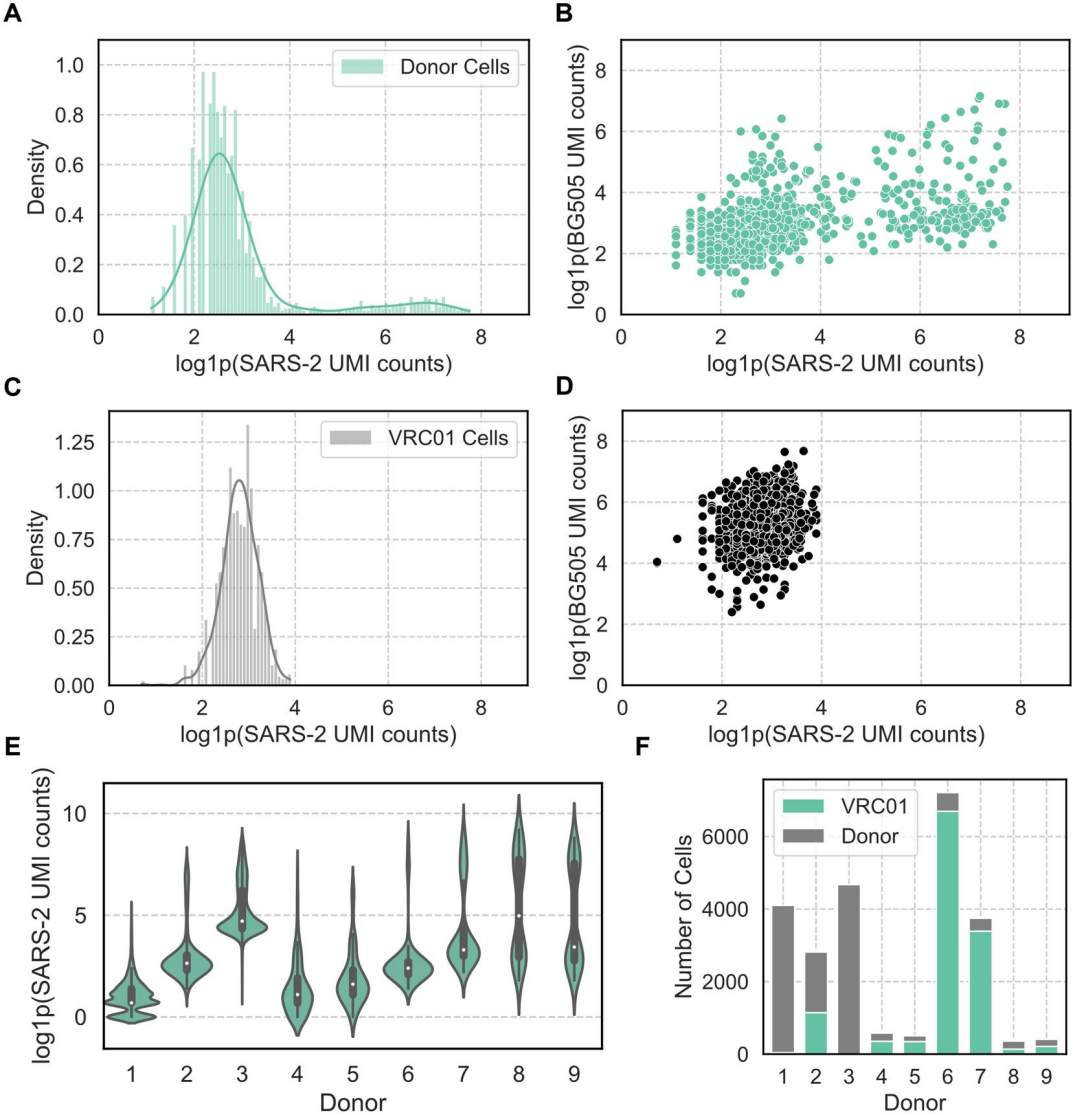
Characterization of UMI noise in LIBRA-seq data donors and negative control cells. Distributions of SARS-CoV-2 spike (SARS-2) UMI counts are shown as histograms with kernel density estimates (KDEs) for (A) donor B cells and (B) VRC01 negative control cells in Donor 2. (C) Plotting BG505 (HIV-1) UMI counts against SARS-CoV-2 UMI counts for Donor 2. (D) BG505 (HIV-1) UMI counts against SARS-CoV-2 UMI counts for Donor 2 VRC01 cells. (E) Violin plot comparing distributions of SARS-CoV-2 UMI counts for B cells across donors. (F) Stacked counts of donor B cells and VRC01 cells for each donor.

Comparing the distribution of antigen UMI counts in donor B cells to VRC01 negative control cells provides a clear visualization of noise for a given sample. In the donor cells (Fig. 2A), a bimodal distribution is seen with one component of lower counts and a second distribution with larger variance at higher UMI counts. In VRC01 cells from the same sample (Fig. 2B), there is a single distribution which appears similar to the lower count distribution observed in the donor cells, suggesting that the antigen UMI counts in the VRC01 cells are representative of noise in the donor cell under the assumption that comparable numbers of UMI reads are recovered for both the VRC01 and donor cells. The second component in the Donor cells, which is not recapitulated in the negative control, is assumed to represent true signal from binding interactions between the B cells and antigen.

A similar bimodal distribution is also seen in the relationship between the SARS-CoV-2 UMI counts and the UMI counts for BG505, which serves as a negative control (Fig. 2C). For Donor 2, there is a positive correlation (Spearman’s *r* = 0.48) between these distributions. In the complete absence of noise, we would expect this correlation to be 0, but this positive correlation implies that there is some degree of non-specific (promiscuous) binding between the BCRs and antigens as very few BCRs should bind to both HIV-1 Env and SARS-CoV-2 spike. The VRC01-expressing negative control cells exhibit more specificity, with a Spearman correlation of 0.21 between BG505 and SARS-CoV-2, suggesting that the BCRs may be responsible for non-specific binding rather than antigen stickiness. While we do not attempt to explore the source of this noise or the binding to BG505 any further in this manuscript, the relationship between SARS-CoV-2 and BG505 UMI counts further motivates the need to filter out false positive UMI counts. Despite the lower correlation between these UMI count distributions for VRC01 cells, Fig. 2D emphasizes that even with an antibody well known to be highly specific to HIV-1, there is a distribution of inflated UMI counts captured for SARS-CoV-2 that represents technical noise in the sample.

The structure of UMI signal and noise is complicated by the variance of distributions across donors (Fig. 2E). For most of the samples, there was a clear visual separation of multiple distributions for SARS-CoV-2 UMI counts (Supplementary Fig. S1). The distributions of SARS-CoV-2 UMI counts in VRC01 cells, representing noise (Supplementary Fig. S2), also showed variation across donors. This variation implies that a single threshold for UMI signal/noise may not be sufficient for filtering across datasets, emphasizing the need for a more sophisticated approach to identifying noise and establishing biologically meaningful thresholds to indicate binding. Sample-to-sample variation was also apparent in the differences observed in the ratio of donor: VRC01 cells (Fig. 2F). The impact of VRC01 on model fitting and accuracy is explored later, as some donors display very low counts of VRC01, with Donor 3 representing an extreme where zero VRC01 cells were recovered.

### 2.2 Algorithm

**Figure 3.**
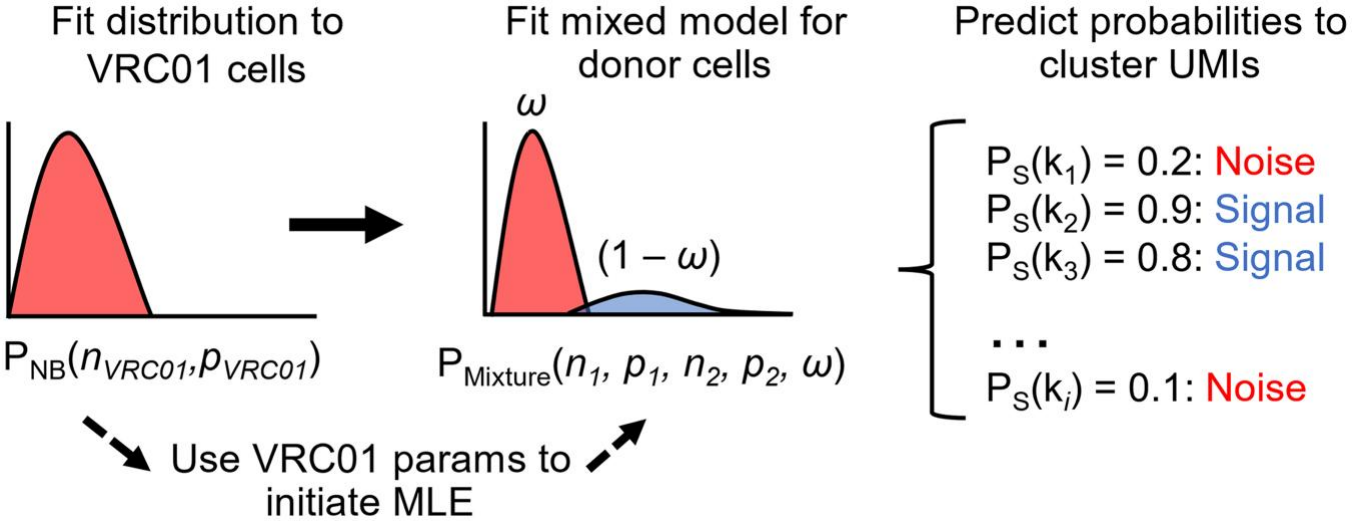
Schematic of mixture model pipeline for predicting noise. First, a distribution is fit to the VRC01 UMI count distribution for SARS-CoV-2 using an MLE. Then, these parameters are used to initialize an MLE to fit a mixed distribution to the SARS-CoV-2 UMI count distribution for the donor B cells. The probability mass function of the mixed distribution is used to generate probabilities for the SARS-CoV-2 UMI counts (*k*) for all cells (*i*) in a donor. Finally, the UMI count is then assigned to one of the components based on a threshold for P_S_ and labeled as either “signal” or “noise.”

The algorithm described here represents a custom mixture model approach using negative binomial distributions to cluster experimental UMI counts. After separating the VRC01 cells from the real donor B cells, the following pipeline was applied to the distribution of counts for each antigen within each donor separately. This approach consists of three steps, which are visualized in Fig. 3. First, a maximum likelihood estimator (MLE) was used to fit a negative binomial distribution to the UMI counts associated with the VRC01 negative control cells.

The probability mass function (PMF) for the negative binomial distribution for a UMI count *k* (1) is described by the parameters *nN* and *pN* (Virtanen *et al.* 2020).

(1) PNB(k|nN, pN)=k+nN−1nN−1pNnN(1-pN)k

The parameters from the noise distribution are then used to initialize step two, where a negative binomial mixture model is fit to the UMI count distribution for the donor cells (2). The mixed distribution consists of two negative binomial components and a weight ω to account for the imbalanced cell counts between the number of VRC01 cells and B cells for different donors. The parameters for this mixed distribution are estimated with an MLE, using the log of the sum of exponentials to minimize the negative loglikelihood (NLL) of the component mixture.

(2) $$P_{\mathrm{Mixture}}\left(k|n_{N},{\;p}_{N},n_{S},{\;p}_{S},\;\omega \right)=\omega P_{\mathrm{NB}}\left(n_{N},{\;p}_{N}\right)+\left(1-\omega \right)P_{\mathrm{NB}}\left(n_{S},{\;p}_{S}\right)$$

Finally, Bayes’ Theorem was employed using the PMFs for each component to calculate the probability for each observation (UMI count) of being in either the signal (*P*_*S*_) or noise (*P*_*N*_) component (3). In the mixed distribution, the components are nonidentifiable, meaning that the component labels are initially arbitrary and are not considered as signal or noise during fitting by MLE. Component labels are assigned *post-hoc*, where the component with the higher median was considered the “Signal” component and the lower median component was labeled as “Noise.” In the results and discussion here, the probabilities referenced will be *P*_S_, the probability of a given UMI count being in the signal component of the mixed distribution.

(3) $$P_{N}\left(k\right)=\frac{\omega P_{\mathrm{NB}}\left({k|n}_{N},{\;p}_{N}\right)}{P_{\mathrm{Mixture}}\left({k|\omega ,\;n}_{N},{\;p}_{N}{,\;n}_{S},{\;p}_{S}\right)}{,\;P}_{S}\left(k\right)=\frac{(1-\omega )P_{\mathrm{NB}}\left({k|n}_{S},{\;p}_{S}\right)}{P_{\mathrm{Mixture}}\left({k|\omega ,\;n}_{N},{\;p}_{N}\;{,\;n}_{S},{\;p}_{S}\right)}$$

The pipeline was implemented using the *Statsmodels* (Seabold and Perktold 2010) and *Scipy* (Virtanen *et al.* 2020) libraries in Python. For step 1, fitting of the noise distribution *P*_NB_, the MLE was initialized with started parameters *p = n = *1. In step 2, fitting of the mixed distribution *P*_Mixture_, the weight was initialized as ω= 0.1. The parameters from the noise distribution were set as *n_N_* and *p_N_*, while *n_S_* and *p_S_* were estimated based on the initial weight guess. Briefly, the weight guess was used to split the UMI count data into two components based on counts above and below a percentile calculated from the weight, then a negative binomial distribution was fit to the lower percentile distribution to estimate starting parameters *n_S_* and *p_S_* for initializing the mixed distribution MLE. For Donor 3, where no VRC01 cells were recovered, this percentile-based approach for estimating starting parameters was used to initialize parameters for both components.

## 3 Results

A critical assumption underlying the use of LSS for binding predictions is that antigen barcodes captured in a droplet represent true binding interactions between the encapsulated B cell and the antigen from which the barcode originated. As discussed earlier, various sources of technical noise can inflate these UMI counts, leading to false positive predictions. The pipeline presented here aims to fit a mixed distribution model to UMI count distributions in the donor cells which can then be used to cluster the counts into noise and signal components, enabling more accurate identification of antigen-specific B cells. This approach is employed separately for each donor and antigen to account for the variability in noise observed across donors.

**Figure 4.**
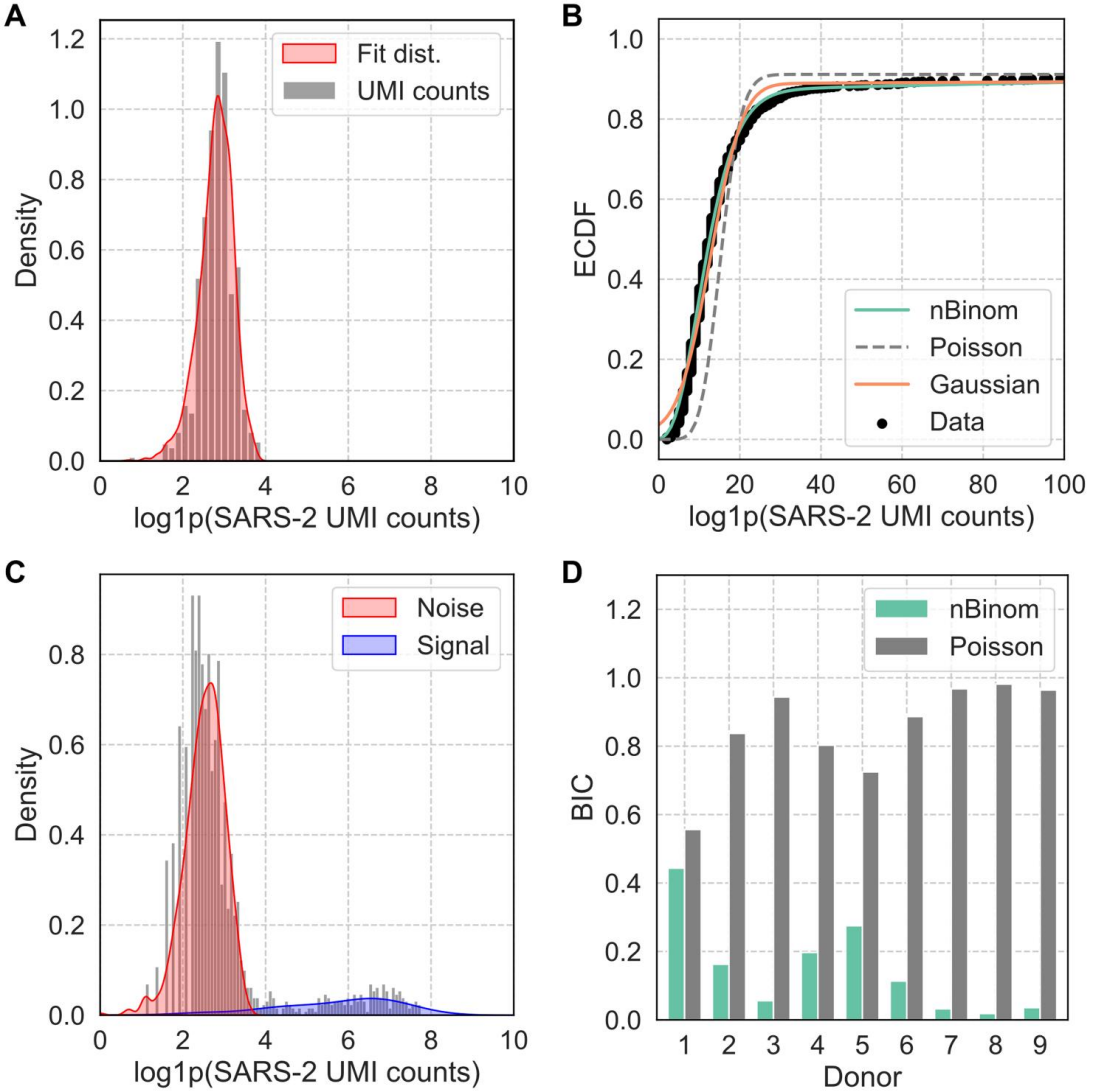
Assessment of fit quality for noise and mixture models for SARS-CoV-2 UMI counts. (A) Visualization of randomly sampled variates from noise distribution fit to SARS-CoV-2 spike (SARS-2) UMI counts captured for VRC01 cells in Donor 2. (B) Comparison of empirical cumulative distribution functions (ECDFs) for mixed distributions of Gaussian, Poisson, and Negative Binomial distributions for Donor 2 SARS-CoV-2 UMI counts. (C) Visualization of KDE for the two components fit for the negative binomial mixture model of SARS-CoV-2 UMI counts for Donor 2. (D) Comparison of Bayesian Information Criteria (BIC) from mixed model fits for each donor.

Counts in single cell data are commonly modeled using a variety of distribution types including Gaussian (Mulè *et al.* 2022) or discrete distributions such as the Poisson and negative binomial distributions (He *et al.* 2021, Fleming *et al.* 2023). In order to determine the best approach for modelling noise in the VRC01 cells, we fit three common distribution types (Supplementary Fig. S3A) and found that the negative binomial distribution (Fig. 4A) had the best fit according to NLL, Akaike Information Criterion (AIC), and Bayesian Information Criterion (BIC). Similarly, after fitting the mixed distributions to the donor cell distributions, we found that the mixed negative binomial model was most representative of the empirical distribution (Fig. 4B) for UMI counts in the donor B cells. The mixed Poisson distribution fit poorly overall, while the mixed Gaussian model appeared to fit very well in the middle range of UMI counts but failed to capture the behavior of the distribution at the extremities for Donor 2. The mixed negative binomial distribution fit with a lower BIC than the mixed Poisson distribution for all donors (Fig. 4D). The mixed Gaussian model was also tested across all donors but consistently failed to converge (Supplementary Fig. S3B).

In addition to BIC, the fit quality of the mixed negative binomial distributions can also be visually confirmed for the VRC01 noise distribution (Fig. 4A) and the mixture model of the donor B cells (Fig. 4C) for Donor 2, along with the rest of the donors (Supplementary Fig. S4). While the distributions are shown here as approximation by kernel density estimates (KDEs), the PMF of the fit distributions are used below for the implementation of the mixture model for denoising. These visualizations emphasize some of the challenges in finding a model that can account for the large variations in UMI distributions observed across donors here. Donor 1, for example, appeared to primarily contain noise with low UMI counts and very few true signal counts. Donors 2 and 6–9 showed relatively well separated distributions of low and high UMI counts, corresponding to the noise and signal components.

### 3.1 Determining quality of signal probability predictions

After fitting the negative binomial mixture model to the donor cell UMI count distribution for SARS-CoV-2 spike, an adaption of Bayes’ theorem was applied to the PMF of the mixed distribution to calculate the probabilities of each UMI count belonging to the signal component. The probabilities output from Bayes’ theorem can then be binned with a threshold to cluster the UMI counts as “signal” or “noise,” similar to a Gaussian mixture model clustering algorithm (Murphy 2012).

Once signal probabilities (*P*_S_) are obtained, the challenge remains to establish a biologically relevant threshold to predict true binding. While this is straightforward with clearly separated distribution components, determining a clustering threshold is more challenging for donors where the two distributions overlap. An unbiased threshold can be chosen as *P*_S_ = *P*_N_ = 0.5, but this threshold could be modified depending on the application. For example, in antibody discovery it may be advantageous to identify a greater number of potential antibody candidates despite the increased risk of false positives, while machine learning applications would benefit from more conservative thresholds to reduce false positives.

**Figure 5.**
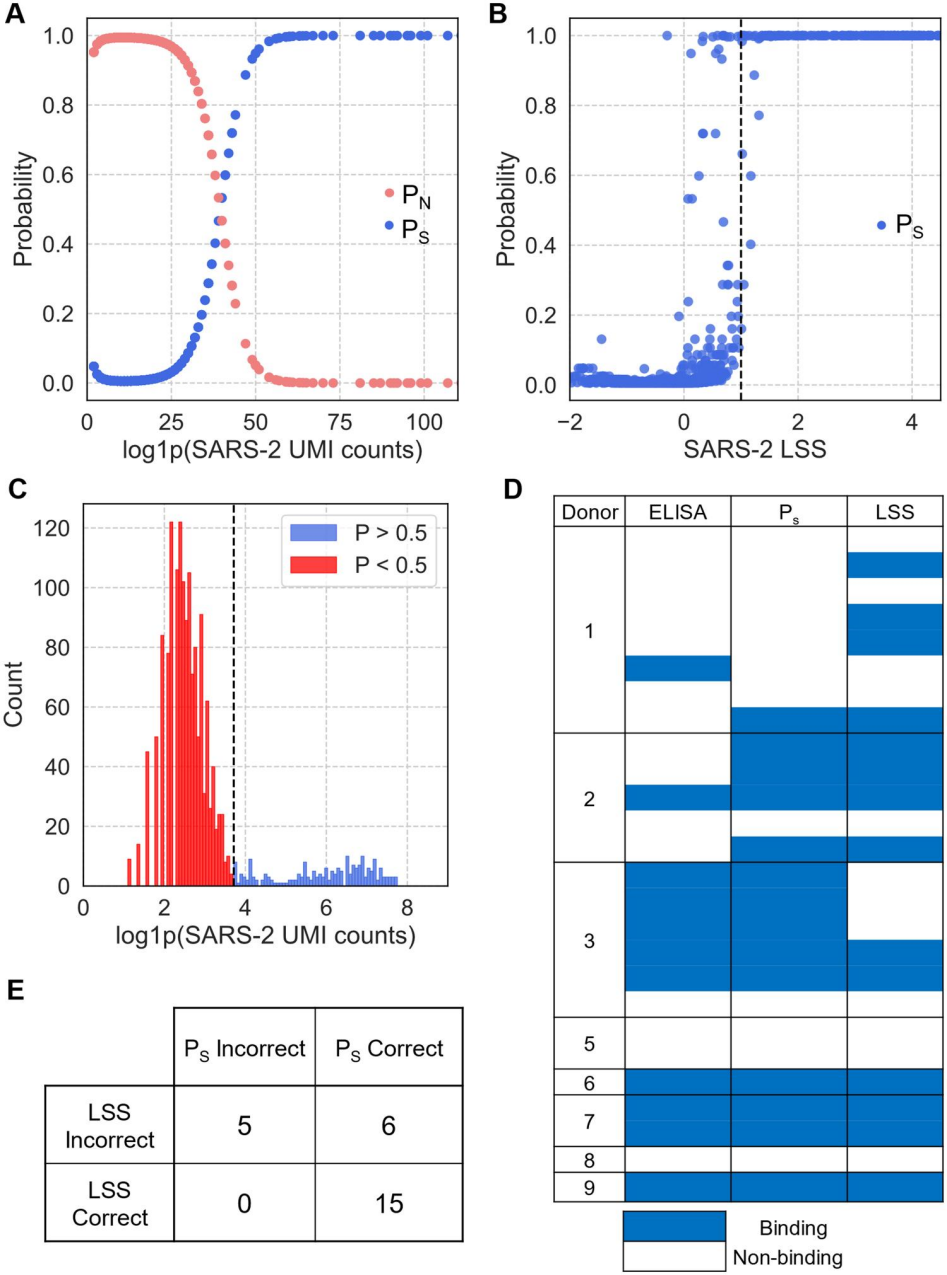
Model output probabilities match *in-vitro* validation by ELISA. After model fitting, the mixture model was used to cluster SARS-CoV-2 spike (SARS-2) UMI counts into the signal and noise components. (A) Plotting *P_S_* against UMI counts for Donor 2 shows the inflection point between components. (B) Plotting *P_S_* against LSS for Donor 2 with dotted lines representing the recommended thresholds for each. (C) Histogram of log1p transformed UMI counts, with labels assigned based on a *P_S_* threshold of 0.9. (D) Absorbance (AU450) values from ELISA micro-expression for recombinantly expressed antibodies compared to LSS and *P_S_* predictions. Binned values are shown based on the following thresholds: LSS ≥1, PS ≥0.9, AU450 ≥ 1. (E) Confusion matrix comparing *P_S_* to ELISA values.

In Fig. 5A, we can see that a sigmoidal curve is formed when comparing the signal probabilities to the antigen UMI counts. Typically, a LSS threshold of 1 is used to classify binding, based on experimental observations during the initial development of the method. In Fig. 5B we see that applying this LSS threshold alone includes a number of probabilities below 0.5 which could result in false positive binding assignments when considering probability alone. Since this is a probabilistic model that aims to identify true UMI counts, rather than directly predicting binding, we recommend using the calculated signal probability in conjunction with the standard LSS threshold to yield the highest confidence predictions. For Donor 2 (Fig. 5C), we can see that the component assignments from clustering intuitively aligned with the anticipated signal/noise division that we would expect based on the VRC01 distribution shown previously in Fig. 2.

### 3.2 *In vitro* validation of binding predictions

In the LIBRA-seq study, binding predictions by LSS were tested *in vitro* by recombinantly expressing the antibodies and performing ELISA (Abu-Shmais *et al.* 2024). We compared these results to predictions made by the signal probabilities (*P*_S_) generated by the mixture model here to determine the accuracy of the model and see how it compares to the standard LSS. For the donors selected here, there were a total of 26 antibodies validated for binding to SARS-CoV-2 spike. LSS accurately predicted binding for 14/26 (54%) antibodies, while the mixture model accurately predicted binding for 20/26 (77%) antibodies based on *P*_S_ (Fig. 5D). This increase in performance was mainly due to improved predictions in Donors 1 and 3, while predictions for all other donors matched the LSS. Importantly, there were no instances where the LSS was correct and *P*_S_ was wrong (Fig. 5E), meaning that the probabilities always matched or improved predictions. In order to predict binding using LIBRA-seq, a fundamental assumption is that UMI counts represent binding, although this relationship may not always be linear or correlative. In many cases where both LSS and *P*_S_ incorrectly predicted, the UMI counts do not appear linked to binding at all, with one example where an antibody bound in ELISA despite only yielding three UMI counts for SARS-CoV-2 spike (Supplementary Fig. S5). Correcting discrepancies such as this is outside the scope of this model, as these are clearly not due to UMI count inflation alone and could result from technical issues during any of the experimental steps in the pipeline.

### 3.3 Necessity of VRC01 negative control cells

The pipeline described here leverages the VRC01 negative control cells included in the LIBRA-seq study (Abu-Shmais *et al.* 2024), providing a data-driven means for introducing bias to model fitting. While this negative control was important for understanding the structure of noise in this dataset, the inclusion of such cells increases cost and labor and can also detract from the total recovery rate of donor B cells for each sequencing run. However, we have shown that the mixture model for noise clustering was still able to enhance LSS predictions for Donor 3, where no VRC01 cells were recovered. To further support the possibility of using this approach in the absence of VRC01, the pipeline was tested on all donors without inclusion of bias from VRC01 for the mixture model MLE. For this approach, the mixed distribution parameters were initialized using the strategy previously described for Donor 3, where mean and variance were estimated from an initial weight guess. We found that *P*_S_ yielded the same predictions as the model using VRC01 for Donors 2–9 but performed worse for Donor 1 (Supplementary Fig. S6A), where there were very few cells with high SARS-CoV-2 UMI counts (Fig. 1E). Without VRC01, we observed that the mixture model overestimated the prevalence of signal, leading to a higher false positive rate (Supplementary Fig. S6B and C). This emphasizes the usefulness of the negative control cells for samples with lower overall recovery against an antigen of interest, where it may be more difficult to differentiate signal from noise in the UMI count distribution.

To further elucidate the impact of the VRC01 negative controls on model fitting, bootstrapping with replacement was performed with and without the VRC01 bias. In the absence of the VRC01 bias, the percentile-based parameter initiation approach was used, as described above. More variability in parameter estimation was observed for Donor 1 (Supplementary Fig. S6D and E), with a higher mean and variance in the distribution of *ω* without the VRC01 bias, implying that this model overestimated the weight of signal in the mixture. We also found that the unbiased fit, without VRC01, led to a higher SEM for predictions (*P*_S_) in 6/8 donors (Supplementary Fig. S6F). Together, these observations reinforce the idea that the use of VRC01 negative control cells to bias the initiation of the mixture model fit can reduce model variability and improve prediction accuracy in some cases. However, we have also shown that this mixture model denoising approach can yield high accuracy predictions without the need for VRC01 cells in many cases, especially when the signal and noise components within the distribution of SARS-CoV-2 UMI counts are well separated.

## 4 Discussion

Identification of noise in LIBRA-seq UMI count distributions is critical for improving antigen binding predictions for downstream applications such as antibody discovery and machine learning technologies. In this study, we present a method for clustering antigen UMI counts into signal and noise components with a negative binomial mixture model. This approach provides improved antigen binding predictions across multiple samples when combined with the standard scoring method (LSS), despite heterogeneity in data size and noise structure.

In the context of the data presented here, we found that the noise distribution within the VRC01 negative control cells was representative of the noise within the bimodal distribution of SARS-CoV-2 UMI counts in donor cells, which was used accordingly in our approach to separate out noise from signal. The Ramos cell line used to express VRC01 for this negative control, however, can be sensitive to handling and recovery can be poor, as seen here with Donor 1 data only containing 43 VRC01 cells, and Donor 3 recovering zero. Additionally, such controls were not included in previously published LIBRA-seq experiments and could limit the application of this method. Notably, we found that the mixture model approach without bias from the negative control cells can work equally as well in cases where the UMI counts form a distinct bimodal distribution. While this data structure may not necessarily be present in some biological contexts, e.g. cells collected from vaccinees or infected individuals, our data shows potential for use of the mixture model approach for denoising without need for the VRC01 negative control cells, enabling its application to a much wider range of samples.

A limitation of the mixture model denoising approach described here is the data size required for accurate clustering. The size threshold for choosing samples initially was somewhat arbitrary since it is difficult to quantify the exact number of cells needed without a much larger *in vitro* validation dataset. In an attempt to estimate the necessary data size for our approach to work, bootstrapping with downsampling was performed (Supplementary Fig. S7). Donor cells were downsampled across a range of total B cell counts in order to estimate model stability. As shown in Supplementary Fig. S7, the coefficient of variation for the BIC of the mixed distribution fit begins to increase rapidly for most donors once the sample size drops below 300 total donor B cells, suggesting a decrease in model fit at low sample sizes. Similarly, the variation of *P*_S_ increases below 300 cells, showing an increased uncertainty in model predictions (Fig. S7B). Based on this bootstrapping analysis, Donor 1 appeared to be most sensitive to decreased sample size. While this may be surprising since Donor 1 had high overall cell counts, it also had the lowest proportion of cells with SARS-CoV-2 UMI counts >10 (7.14%). Due to the previously discussed variation is UMI count distributions, the necessary sample size may ultimately depend on the separation and ratio of noise/signal components, but bootstrapping suggests that a total cell count of at least 300 B cells is necessary for stable model fitting and low prediction error. The standard error of the mean signal probabilities for 100 iterations of bootstrapping with resampling are shown in Supplementary Fig. S7C. Donor 1 displayed the highest error of 2.2% SEM, likely due to the low ratio of high SARS-CoV-2 UMI counts relative to the noise distribution, as mentioned above. For all other donors, the error in *P*_S_ was <1%, suggesting that the model fit reliably and was not sensitive to cell sampling.

## 5 Conclusion

Overall, we have shown that our negative binomial mixture model approach for denoising LIBRA-seq data is able to accommodate sample-to-sample variability and improve antigen-binding predictions for B cells across nine individual samples collected from separate donors. This development will improve the ability of LIBRA-seq to identify antigen-specific B cells and contribute to providing more reliable datasets for future machine learning based approaches to predicting antibody-antigen binding as the corpus of single-cell B cell sequencing data continues to grow.

## Supplementary Material

vbae170_Supplementary_Data

## Data Availability

The data underlying this article are available at https://github.com/IGlab-VUMC/mixture_model_denoising.
