# Supplementary material for: Negative binomial mixture model for identification of noise in antibody-antigen specificity predictions from single-cell data
Source: Bioinform Adv. 2024 Dec 4;4(1):vbae170. doi: 10.1093/bioadv/vbae170 (PMC11631427; doi:10.1093/bioadv/vbae170)
Supplement: vbae170_Supplementary_Data [file vbae170_supplementary_data.pdf]

	Donor ID								
**Cells**	**1**	**2**	**3**	**4**	**5**	**6**	**7**	**8**	**9**
**SARS-CoV-2**	253	1182	4712	48	39	276	356	215	181
**Donor Total**	4100	1690	4721	229	160	523	361	226	199
**VRC01**	43	1155	0	357	351	6766	3424	139	214

Table S1. Cell counts from LIBRA-seq Atlas experiments.
Cell counts shown for donor cells with >10 SARS-2 UMI counts, total donor B cells, and total VRC01 Ramos negative control cells following preprocessing.

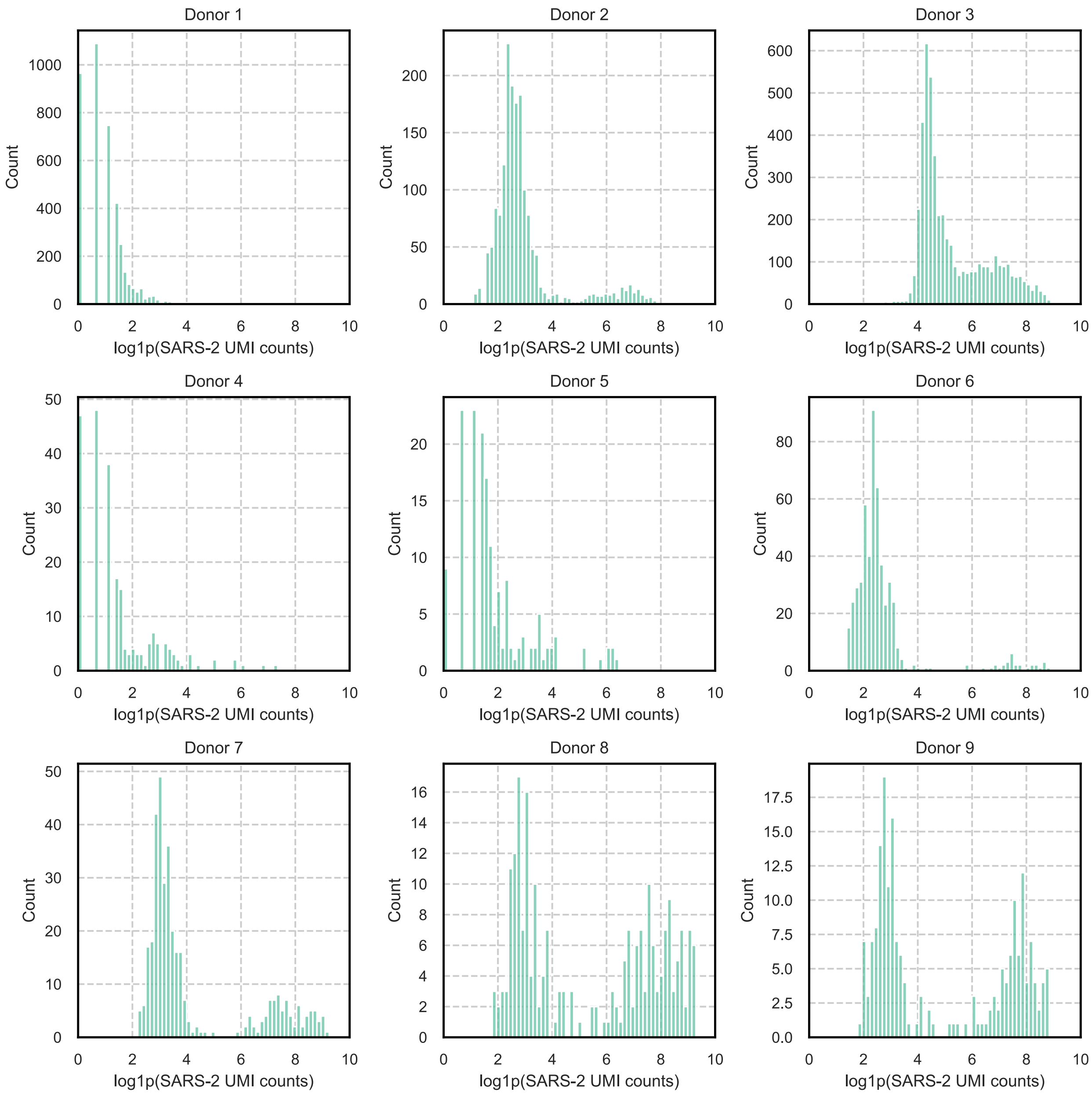

Figure S1. SARS-CoV-2 UMI count distributions across donors
Histograms representing distributions SARS-CoV-2 spike (SARS-2) UMI counts captured for donor B cells in each donor.

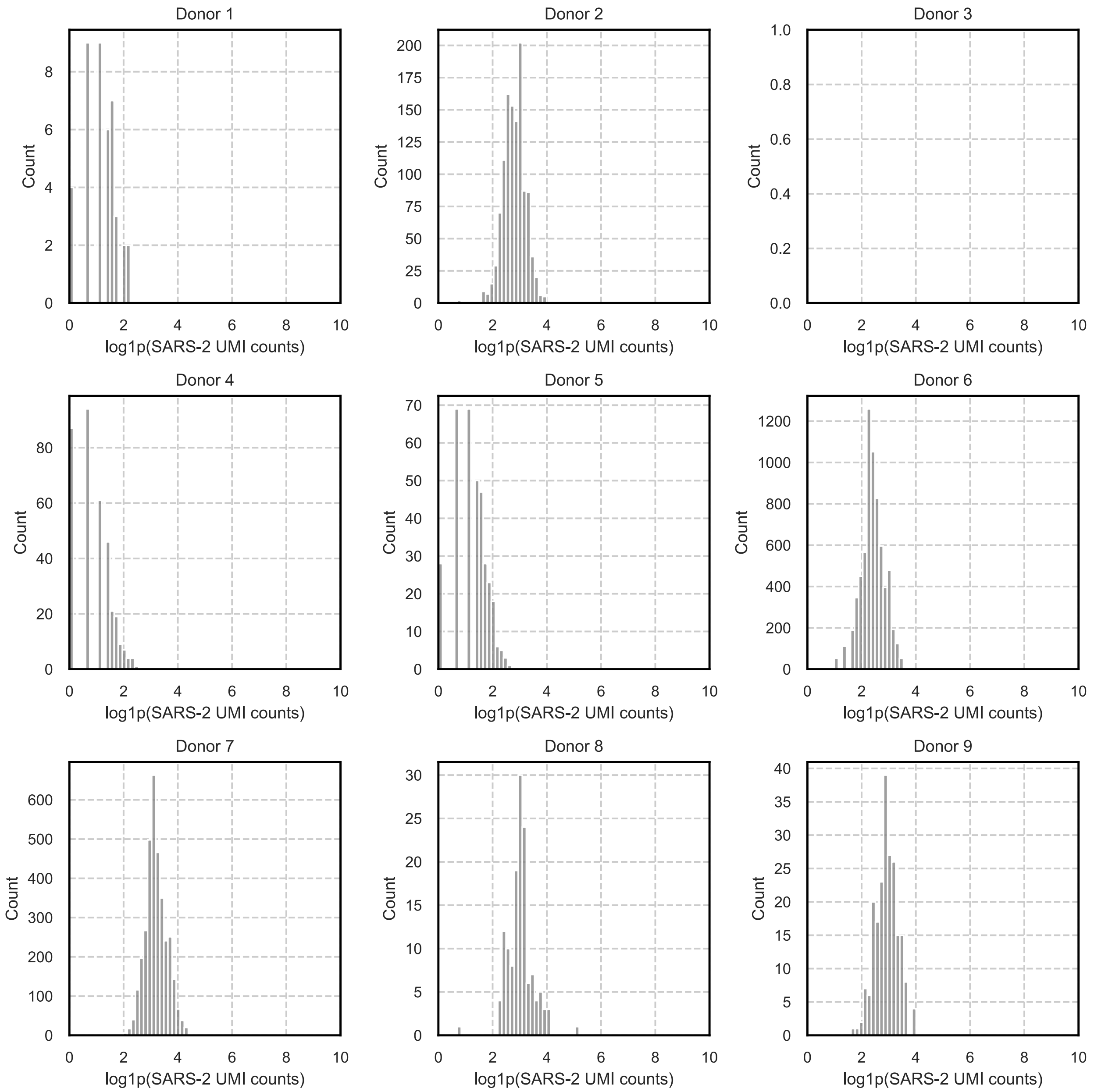

Figure S2. SARS-CoV-2 UMI count distributions for VRC01 across donors Histograms representing distributions SARS-CoV-2 spike (SARS-2) UMI counts captured for VRC01 expression Ramos cells in each donor.

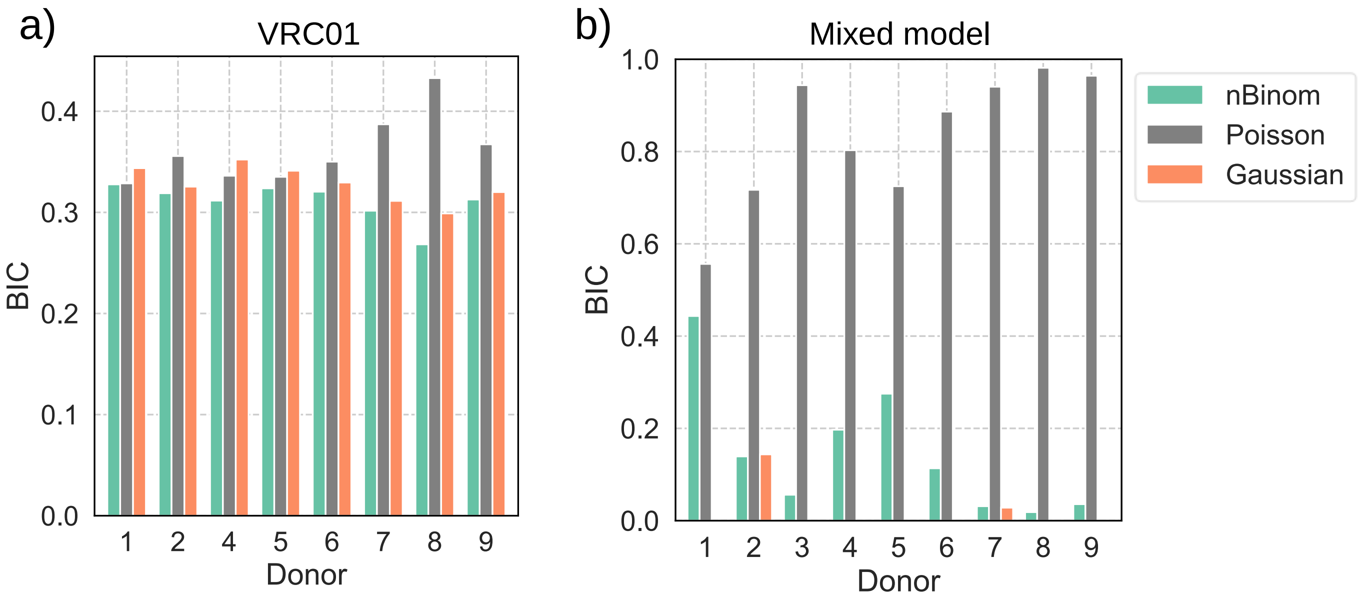

Figure S3. Fit quality metrics for SARS-CoV-2 UMI distributions across donors
a) Comparison of BIC for negative binomial, Poisson, and Gaussian distributions fit to SARS-CoV-2 spike (SARS-2) UMI counts in VRC01 cells. b) Comparison of BIC for mixture models of binomial, Poisson, and Gaussian distributions fit to SARS-CoV-2 UMI counts in donor cells.

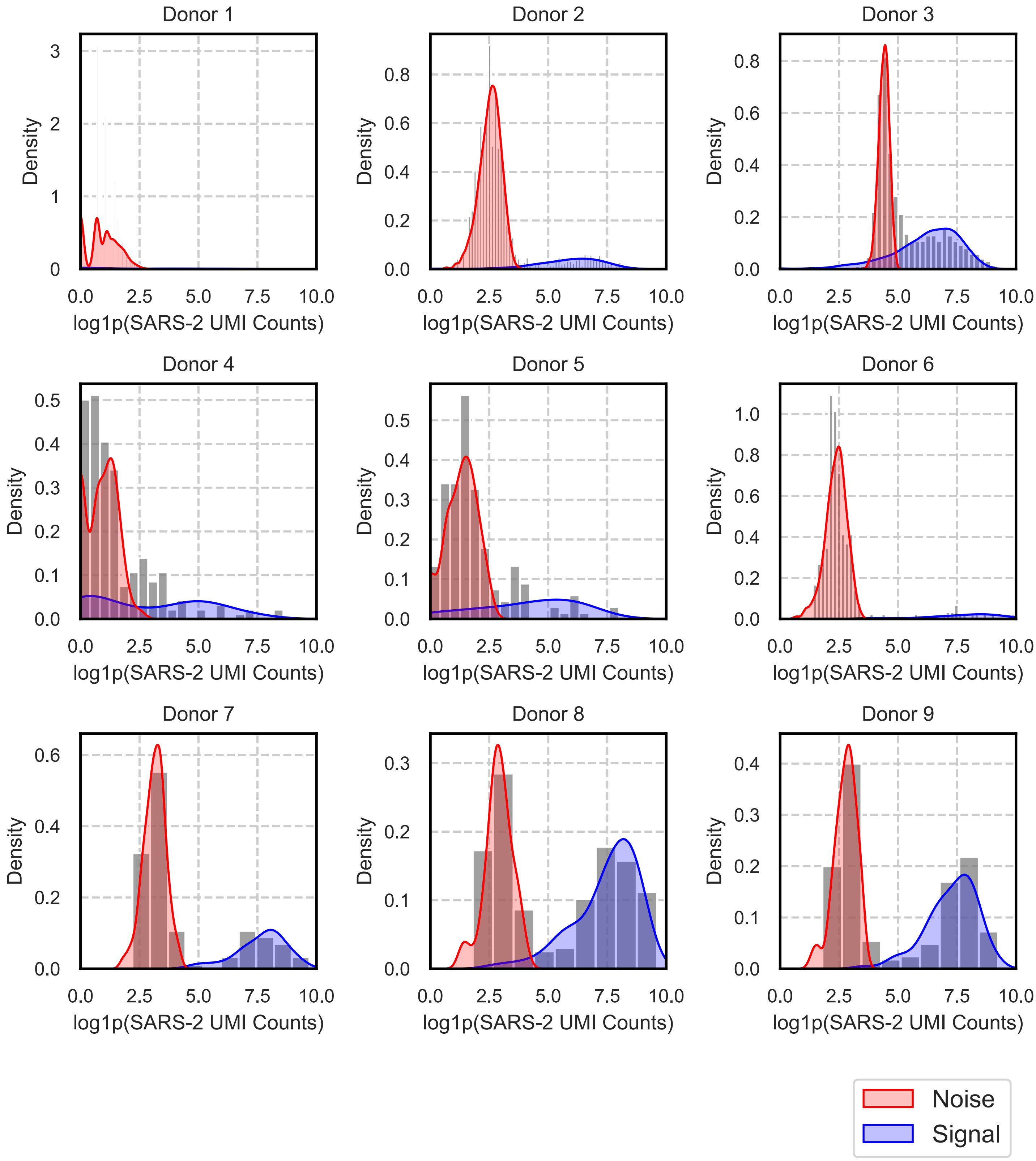

Figure S4. Mixed model fit for all samples for SARS-2
a) Visualization of KDE for the two components fit for the negative binomial mixed distributions of SARS-CoV-2 spike (SARS-2) UMI counts for all 9 donors.

a)

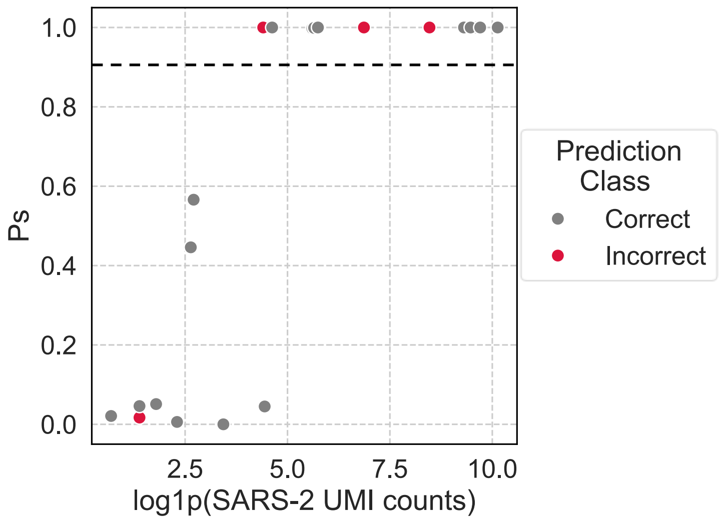

b)

ELISA	P_S	Prediction Class	SARS-2 UMI counts
3.245	0.017	False negative	3
0.089	1	False positive	81
0.078	1	False positive	87
0.149	1	False positive	958
0.136	1	False positive	4752

Figure S5. Relationship between Ps and SARS-2 UMI counts for validated cells.
A) Scatterplot showing prediction probabilities plotted against the log(x+1) for SARS-2 UMI counts. Black dotted line represents the threshold used for binning predictions. Points colored by correct or incorrect predictions based on ELISA. B) Table comparing ELISA absorbance values against Ps, prediction class, and UMI counts.

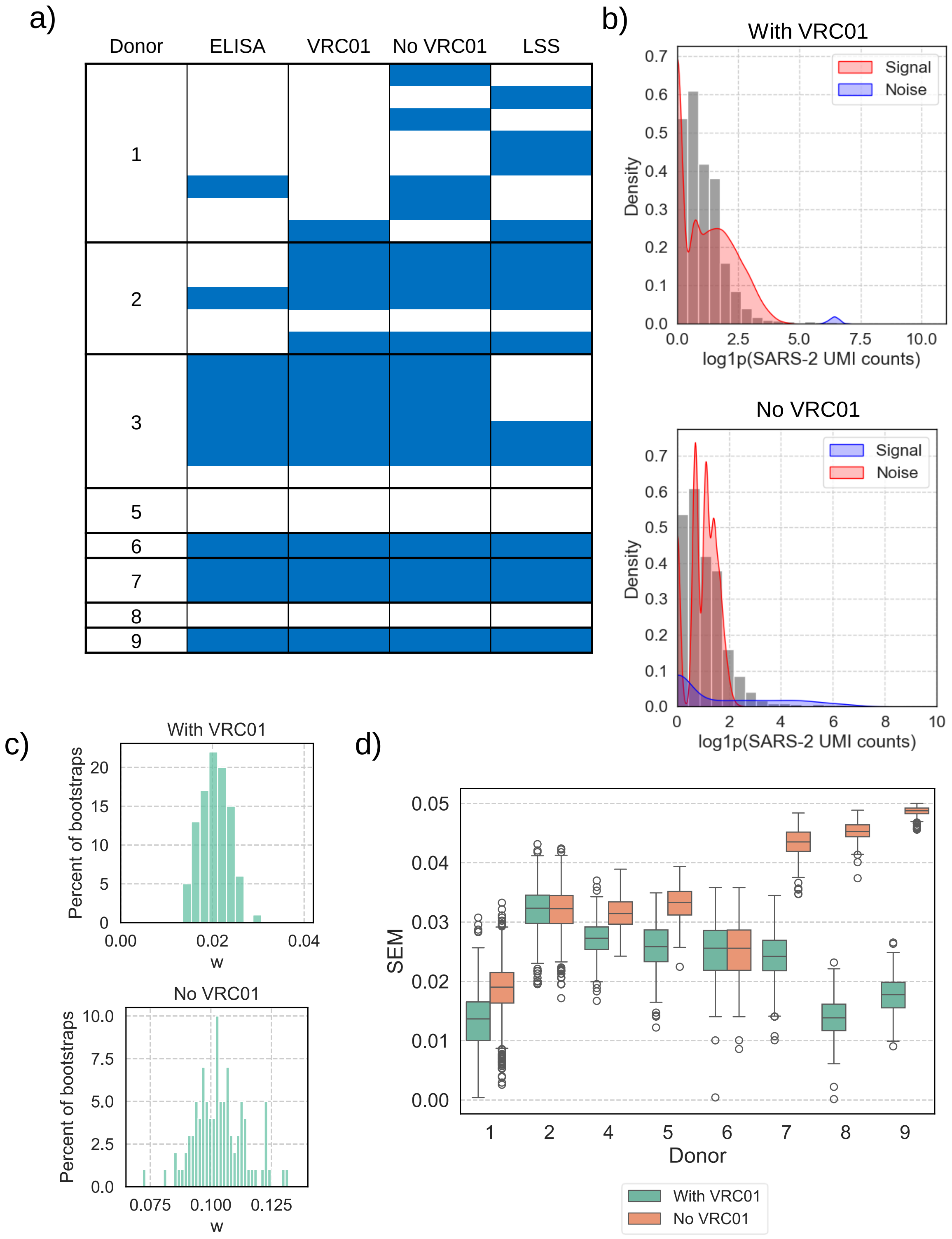

Figure S6. Model can work in absence of negative control with adequate amount of data. A) Comparison of ELISA Absorbance (AU450) values from ELISA micro-expression for recombinantly expressed antibodies with Ps and LSS predicting binding to SARS-CoV-2 spike using the mixture model fit to all donor cells for each donor, with and without inclusion of VRC01. Binned values are shown based on the following thresholds: LSS ≥ 1, PS ≥ 0.9, AU450≥ 1. B) Visualization of KDE for mixture models fit with and without VRC01 bias for Donor 1 cells. C) Histogram showing distribution of ω values from bootstrapping fit for mixture model with and without VRC01. Model was fit 100 times using resampling with replacement for UMI counts. D) Standard error of mean for Ps from bootstrapping with and without VRC01 bias.

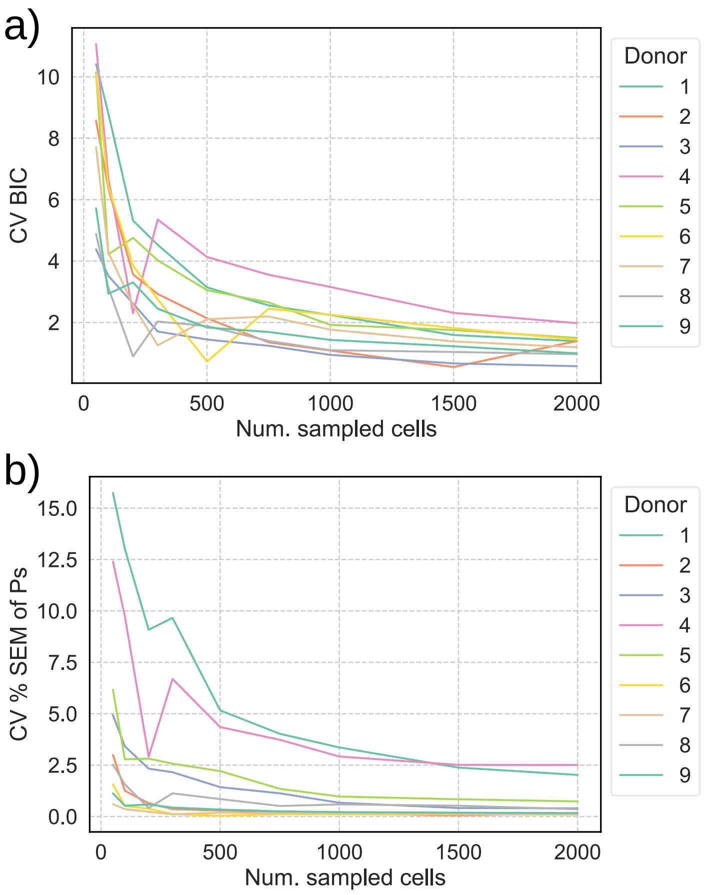

c)

Donor	Ps SEM (%)
1	2.239
2	0.022
3	0.022
4	0.406
5	0.232
6	0.048
7	0.081
8	0.071
9	0.071

Figure S7. Bootstrapping and uncertainty quantification
a). b) Line plots representing relative variance of noise and signal component means across series of downsampled datasets for Donor 2 SARS-CoV-2 UMI counts in each donor. 50 iterations were performed per downsampled data set per donor, with resampling. c) Mean percent standard error across all cells for each donor after 100 iterations of bootstrapping with resampling.
